# Effect of emergency physician-operated emergency short-stay ward on emergency department stay length and clinical outcomes: a case-control study

**DOI:** 10.1186/s12873-023-00813-x

**Published:** 2023-05-12

**Authors:** Sean Moon, Taegyun Kim, Heesu Park, Hayoung Kim, Jieun Shin, Yun Seong Park, Gaonsorae Wang

**Affiliations:** 1 Department of Emergency Medicine, Seoul National University Hospital, 101, Daehak-ro, Jongno-gu, Seoul, 03080 Republic of Korea; 2 Department of Emergency Medicine, Seoul National University College of Medicine, 103, Daehak-ro, Jongno-gu, Seoul, 03080 Republic of Korea; 3 College of Medicine, Seoul National University Hospital, Seoul, Republic of Korea; 4 Disaster Medicine Research Center, Medical Research Center, Seoul National University, 103, Daehak-ro, Jongno-gu, Seoul, 03080 Republic of Korea; 5 Department of Critical Care Medicine, Seoul National University Hospital, 101, Daehak-ro, Jongno-gu, Seoul, 03080 Republic of Korea

**Keywords:** Emergency Short-Stay ward, Emergency Department, Length of stay

## Abstract

**Background:**

We hypothesized that an emergency short-stay ward (ESSW) mainly operated by emergency medicine physicians may reduce the length of patient stay in emergency department without expense of clinical outcomes.

**Methods:**

We retrospectively analysed adult patients who visited the emergency department of the study hospital and were subsequently admitted to wards from 2017 to 2019. We divided study participants into three groups: patients admitted to ESSW and treated by the department of emergency medicine (ESSW-EM), patients admitted to ESSW and treated by other departments (ESSW-Other) and patients admitted to general wards (GW). The co-primary outcomes were ED length of stay and 28-day hospital mortality.

**Results:**

In total, 29,596 patients were included in the study, and 8,328 (31.3%), 2,356 (8.9%), and 15,912 (59.8%) of them were classified as ESSW-EM, ESSW-Other and GW groups, respectively. The ED length of stay of the ESSW-EM (7.1 h ± 5.4) was shorter than those of the ESSW-Other (8.0 ± 6.2, P < 0.001) and the GW (10.2 ± 9.8, P < 0.001 for both). Hospital mortality of ESSW-EM (1.9%) was lower than that of GW (4.1%, P < 0.001). In the multivariable linear regression analysis, the ESSW-EM was independently associated with shorter ED length of stay compared with the both ESSW-Other (coefficient, 1.08; 95% confidence interval, 0.70–1.46; P < 0.001) and GW (coefficient, 3.35; 95% confidence interval, 3.12–3.57; P < 0.001). In the multivariable logistic regression analyses, the ESSW-EM was independently associated with lower hospital mortality compared with both the ESSW-Other group (adjusted P = 0.030) and the GW group (adjusted P < 0.001).

**Conclusions:**

In conclusion, the ESSW-EM was independently associated with shorter ED length of stay compared with both the ESSW-Other and the GW in the adult ED patients. Independent association was found between the ESSW-EM and lower hospital mortality compared with the GW.

## Background

Emergency department length of stay and crowding can occur when there is shortage of resources such as bed space and staffing. ED length of stay has been known to affect clinical outcomes of ED patients as well as ED indicators [1]. In some countries, governments limit patient stay at EDs by government policy [2–4]. The Emergency Medical Service Act was newly enacted and implemented in the Republic of Korea, which restricts the number of patients staying for more than 24 h less than 5% from 2017 [5].

Emergency medicine (EM) is a branch of medicine which is specialized for medical emergency requiring immediate response, including initial resuscitation and stabilization in patients in EDs. EM physicians mostly focus on acute and subacute stage of disease in EDs. Therefore, there are concerns that the capability of EM physicians to take care of patients after stabilization could be less competitive compared with that of physicians caring for patients in wards [6, 7].

Emergency short stay ward (ESSW) is one of the strategies for shortening the ED length of stay and for ameliorating subsequent deterioration of patients’ condition [8]. Current studies have shown that ESSW alleviated ED crowding and was associated with less adverse events and a low rate of ICU admission [9–11]. However, few studies have reported on the clinical utility of ESSW mainly operated by EM physicians [7]. We hypothesized that an ESSW mainly operated by EM physicians may reduce the ED length of stay without expense of clinical outcomes.

## Methods

### Study setting

This study was a retrospective observational study from a tertiary academic hospital in Seoul, Republic of Korea. The study hospital is a regional emergency medical center where complicated patients are transferred to and receive definitive treatment at, as well as local emergency patients visit. As the Regional Emergency Medical Center Designation Criteria includes the operation of at least 30 emergency hospital beds designated for patients who visit the regional emergency medical center and require hospital admission, a 30-beds ESSW has been operated in the study hospital as a part of the regional emergency medical center since April 1, 2004.

All the emergency medical institutions are evaluated and graded annually by the National Emergency Medical Center in the Republic of Korea. The Emergency Medical Institution Evaluation Criteria includes mean length of stay in the emergency hospital beds for the regional emergency medical centers, with less than or equal to 72 h as the highest score. Based on the Emergency Medical Institution Evaluation Criteria, the ESSW of the study hospital allows the patients to stay for three days at maximum in principle. For there is a shortage of ward all the time in the study hospital, a large number of patients admitted to the ESSW are transferred to other hospital within several days.

Among patients who visit the emergency room in the study hospital, patients who need short-term observation or emergency procedures are main candidates for the ESSW admission, although there are no stipulated admission or exception criteria for ESSW admission. Critically ill patients, such as those who are hemodynamically unstable and require high-dose vasopressors and/or inotropes, those who are under respiratory failure and are treated with high-flow nasal cannula or noninvasive/invasive mechanical ventilation are not indicated for the ESSW admission. Every admission to the ESSW is confirmed by an EM attending staff and a fourth-year EM resident who are in charge of operating the ESSW according to their own decision. Most patients in the ESSW are admitted to the department of EM and are treated by EM physicians. The EM physicians mainly care for patients with medical diagnosis in the ESSW, and they also treat patients with certain diagnosis such as airway foreign body, intoxication etcetera. Patients with certain department-specific diagnosis are treated by doctors from corresponding departments (e.g., Patients with ischemic stroke are cared by neurologists and patients with mechanical ileus are cared by surgeons.) in the ESSW.

Two first-year residents in EM, a fourth-year resident in EM and an attending staff work for the ESSW. The first-year residents work shifts every 24 h, and the fourth-year resident and the attending staff work in the weekday from 8 a.m. to 6 p.m., mainly supervising the first-year residents. The first-year residents are in charge of primary response and care for the patients admitted to the department of EM in the ESSW.

From September 2017 to the end of the study period, the study ED was staffed by specialists for general surgery, neurology, neurosurgery and orthopedic surgery. A specialist for internal medicine had worked in the ED as well, from September 2017 to December 2018. They treated for the patients corresponding to their own specialties in the ED and the ESSW in the weekday from 8 a.m. to 6 p.m. As for the other patients in the ESSW and the patients in general wards other than the ESSW, first-year residents of corresponding departments care for them primarily.

### Patient selection

For a new electronic health record system was implemented in the study hospital on November 19, 2016, and the coronavirus disease 2019 outbreak became significant around early 2020 in Republic of Korea, we screened patients who had visited the adult emergency department of the study hospital from January 1, 2017 to December 31, 2019. We included patients who visited the adult emergency department of the study hospital who were aged more than or equal to 19 years and who were admitted to wards of the study hospital. Exclusion criteria were admission to any intensive care units directly from the ED, surgery on the day of admission and direct admission bypassing the ED.

### Data collection

We used the clinical data warehouse system of the study hospital for data collection. Collected data are as following: age, sex, date and time of ED visit, date and time of ED discharge, KTAS level, route of ED visit, type of ED visit (medical or non-medical), initial systolic blood pressure, initial diastolic blood pressure, initial heart rate, initial respiratory rate, initial body temperature, initial response, date of admission to ward, date of discharge from ward, ward of admission, discharge type, discharge result, date of any operation, intensive care unit admission days and ED revisit after transfer to other hospital.

### Study groups and outcome measures

We assigned study participants into three groups: patients admitted to ESSW and treated by the department of emergency medicine (ESSW-EM), patients admitted to ESSW and treated by other departments (ESSW-Other) and patients admitted to general wards (GW). The co-primary outcomes were ED length of stay and 28-day hospital mortality. Secondary outcomes were hospital length of stay, type of discharge and intensive care unit admission during the index admission. For significant proportion of patients are transferred to other hospitals after admission to the ESSW, we also analyzed 7-day ED revisit after transfer to other hospitals as a secondary outcome.

### Statistical analysis

Continuous variables were presented as means ± standard deviations and compared using analyses of variance, and categorical variables were presented as n (%) and compared using chi-square tests. For primary outcomes, we performed *post hoc* Student’s t tests and Chi-square tests with Bonferroni correction after analyses of variance or chi-square tests.

Multivariable linear regression analysis and multivariable logistic regression analysis were used to investigate association between predictor variables and outcome variables. Variables used in the multivariable analyses were included when they were statistically significant in univariable analyses. Among the independent variables, systolic blood pressure, heart rate and body temperature were divided into three categories by their own criteria: 90 mmHg and 120 mmHg, 50 beats per min and 100 beats per min and 36 and 38 °C, respectively.

We used a multiple imputation and chained equations technique to handle missing data. As missing proportion of peripheral capillary oxygen saturation value was over 20%, we created a new predictor variable indicating the missingness of it. For working status of the regional emergency medical center specialists was different every year and the presence of the specialists, who care patients of their own specialty in the ESSW, might have had association with the decision to admission to the ESSW, we divided patients into three subgroups according to the year of hospital visit and compared the primary outcomes among patient groups (ESSW-EM, ESSW-Other and GW) in each visit year subgroup. The patients were also stratified according to KTAS levels and the outcomes were compared in each stratified groups. Patients with KTAS level 4 and 5 were combined into one group, for the number of patients with KTAS 5 was extremely small. Two-sided P values less than 0.05 were considered statistically significant. The entire analyses were performed with R version 4.1.2 (R foundation).

## Results

**Fig. 1:**
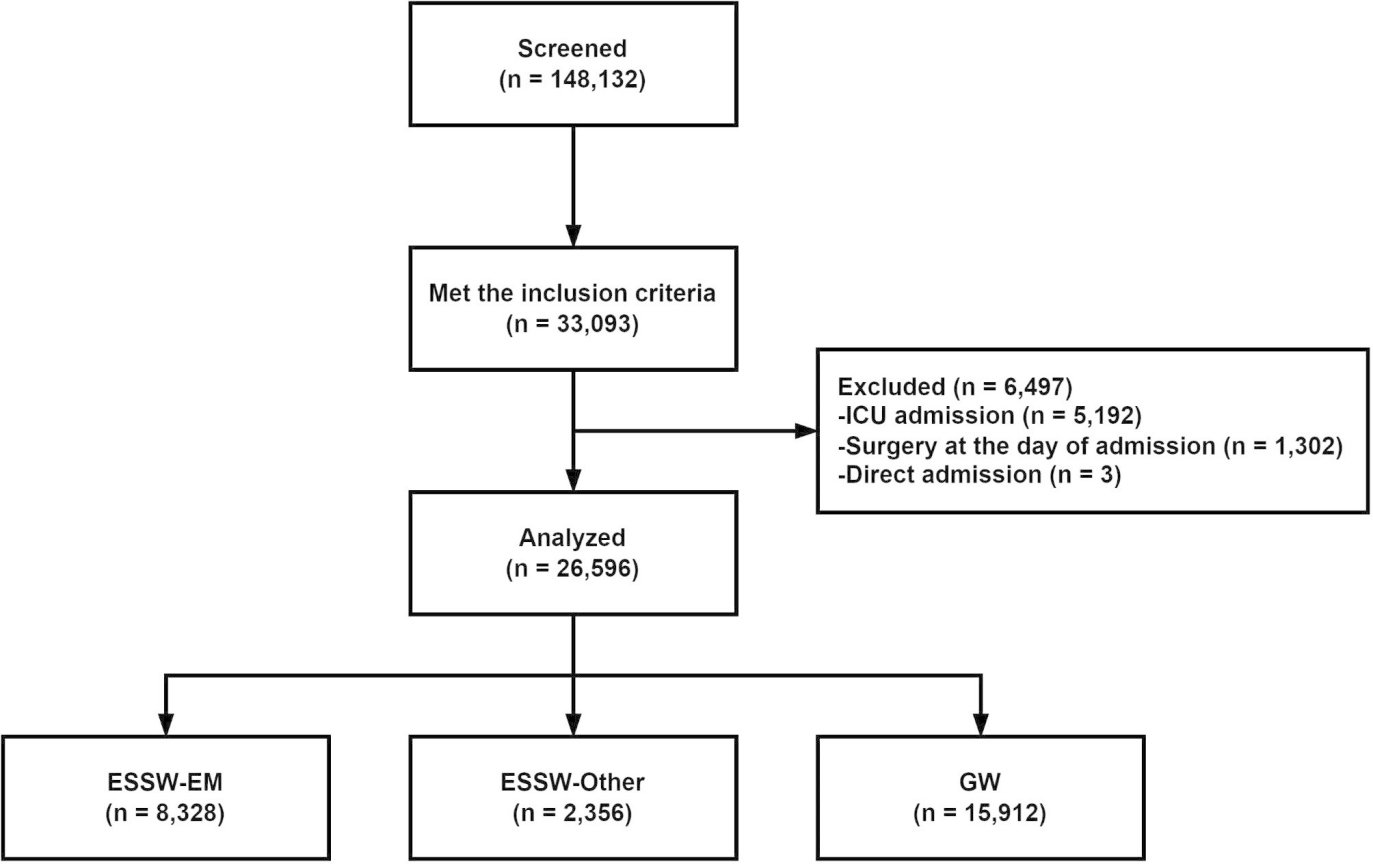
The study flow diagram EM, emergency medicine; ESSW, emergency short-stay ward; GW, general ward; ICU, intensive care unit

**Fig. 2:**
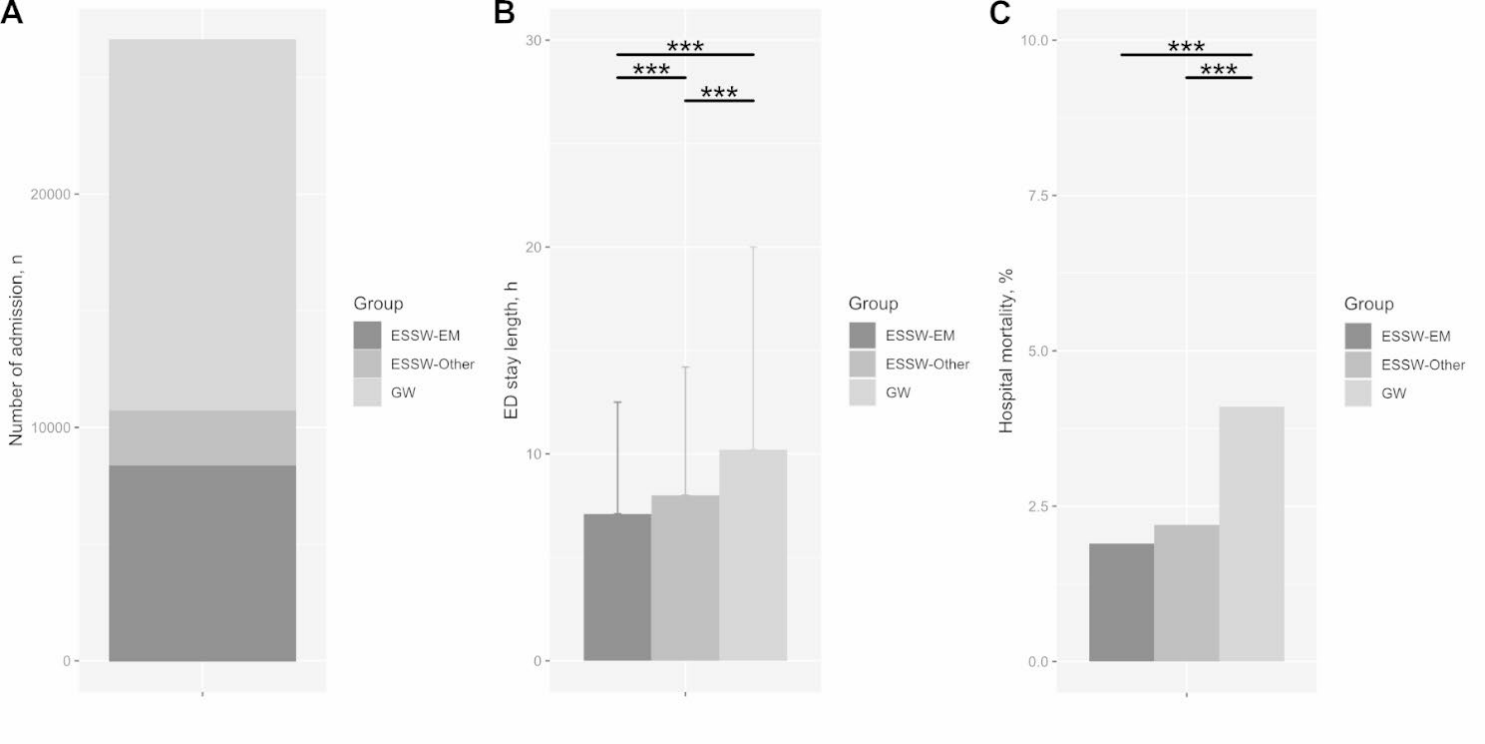
Distribution, ED stay length and hospital mortality according to the study group **2A**: distribution of the participants according to the study group; **2B**: ED stay length according to the study group; **2C**: hospital mortality according to the study group ^***^P < 0.001 ED, emergency department; EM, emergency medicine; ESSW, emergency short-stay ward; GW, general ward

In total, 26,596 patients of 148,132 screened patients were included in the final analysis, and 8,328 (31.3%), 2,356 (8.9%) and 15,912 (59.8%) of them were classified as ESSW-EM, ESSW-Other and GW groups, respectively (Figs. 1 and 2A). Overall, there was significant difference in baseline characteristics among study groups, including age, proportion of male sex, distribution of KTAS levels, visit routes, type of visit, systolic blood pressure, diastolic blood pressure, heart rate, respiratory rate, body temperature, proportion of missing peripheral capillary oxygen saturation, patient response and year of ED visit (Table 1).

**Table 1:** Baseline characteristics according to the initial admission ward

	ESSW-EM	ESSW-Other	GW	*P* value
	(n = 8,328)	(n = 2,356)	(n = 15,912)	
Age, years	66.3 ± 14.1	62.3 ± 15.2	59.7 ± 17.1	< 0.001
Male sex, n (%)	4,860 (58.4%)	1,401 (59.5%)	8,355 (52.5%)	
KTAS level, n (%)				< 0.001
KTAS 1	188 (2.3%)	48 (2.0%)	395 (2.5%)	
KTAS 2	2,023 (24.3%)	442 (18.8%)	3,434 (21.6%)	
KTAS 3	5,932 (71.2%)	1834 (77.8%)	9,862 (62.0%)	
KTAS 4	179 (2.1%)	30 (1.3%)	1,994 (12.5%)	
KTAS 5	6 (0.1%)	2 (0.1%)	227 (1.4%)	
Visit route, n (%)				< 0.001
Direct visit	5,408 (64.9%)	1,415 (60.1%)	9,765 (61.4%)	
Via outpatient clinic	1,151 (13.8%)	481 (20.4%)	2,063 (13.0%)	
Via outside hospital	1,767 (21.2%)	458 (19.4%)	4,078 (25.6%)	
Others	2 (0.0%)	1 (0.0%)	3 (0.0%)	
Unknown	0 (0.0%)	1 (0.0%)	3 (0.0%)	
Type of visit				< 0.001
Medical	8,252 (99.1%)	2,295 (97.4%)	14,622 (91.9%)	
Non-medical	76 (0.9%)	61 (2.6%)	1290 (8.1%)	
Systolic blood pressure, mmHg	139.3 ± 30.6	140.8 ± 29.9	144.4 ± 31.1	< 0.001
Diastolic blood pressure, mmHg	77.4 ± 15.9	79.3 ± 15.6	81.0 ± 16.5	< 0.001
Heart rate, beat per min	91.8 ± 21.1	90.6 ± 19.9	91.0 ± 20.9	0.007
Respiratory rate, breath per min	19.0 ± 3.9	18.8 ± 3.5	18.8 ± 4.0	< 0.001
Body temperature, °C	36.8 ± 1.2	36.8 ± 1.1	36.8 ± 1.0	0.183
SpO2, %	96.4 ± 4.1	96.4 ± 3.9	96.3 ± 4.5	0.631
Missing SpO2 value, n (%)	1686 (20.2%)	542 (23.0%)	4396 (27.6%)	
Patient response, n (%)				< 0.001
Alert	7,914 (95.0%)	2,223 (94.4%)	14,716 (92.5%)	
Response to verbal stimulus	336 (4.0%)	110 (4.7%)	923 (5.8%)	
Response to pain	72 (0.9%)	20 (0.8%)	205 (1.3%)	
Unresponsive	6 (0.1%)	3 (0.1%)	68 (0.4%)	
Year of emergency department visit, n (%)				< 0.001
2017	2,979 (35.8%)	415 (17.6%)	5,055 (31.8%)	
2018	2,525 (30.3%)	1,249 (53.0%)	5,414 (34.0%)	
2019	2,824 (33.9%)	692 (29.4%)	5,443 (34.2%)	

ESSW, emergency short-stay ward; EM, emergency medicine; GW, general ward; KTAS, Korean Triage and Acuity Scale; SpO2, peripheral capillary oxygen saturation

The length of ED stay showed significant difference among the three groups (P < 0.001, Table 2), and the length of ED stay of the ESSW-EM group was significantly shorter than those of both the ESSW-Other group (adjusted P < 0.001, Fig. 2B) and the GW group (adjusted P < 0.001, Fig. 2B). Hospital mortality also showed significant difference among the three groups (P < 0.001. Table 2), however, hospital mortality of the ESSW-EM group was only lower than that of GW group (adjust P < 0.001, Fig. 2C). All the secondary outcomes including hospital day, type of discharge and ICU admission were different among the study groups (P < 0.001 for all three outcome variables, Table 2). The rate of ED revisit within 7 days after transfer to other hospital were similar among the study groups (P = 0.353, Table 3).

**Table 2:** Outcome measures according to the initial admission ward

	ESSW-EM	ESSW-Other	GW	*P* value
	(n = 8,328)	(n = 2,356)	(n = 15,912)	
Primary outcomes				
ED stay length, h	7.1 ± 5.4	8.0 ± 6.2	10.2 ± 9.8	< 0.001
Hospital mortality at day 28, n (%)	156 (1.9%)	51 (2.2%)	656 (4.1%)	< 0.001
Secondary outcomes				
Hospital day, days	6.8 ± 10.0	9.0 ± 12.9	13.9 ± 22.6	< 0.001
Status at day 28, n (%)				< 0.001
Discharged	6,117 (73.5%)	2,008 (85.2%)	13,440 (84.5%)	
Transferred to other hospital	1781 (21.4%)	166 (7.0%)	262 (1.6%)	
Staying at the hospital	274 (3.3%)	131 (5.6%)	1,554 (9.8%)	
Deceased	156 (1.9%)	51 (2.2%)	656 (4.1%)	
ICU admission, n (%)	480 (5.8%)	95 (4.0%)	1,113 (7.0%)	< 0.001

ESSW, emergency short-stay ward; EM, emergency medicine; GW, general ward; ED, emergency department; ICU, intensive care unit

**Table 3:** Emergency department revisit within 7 days after transfer to other hospital

	ESSW-EM	ESSW-Other	GW	*P* value
	(n = 1,781)	(n = 166)	(n = 262)	
Revisit within 7 days, n (%)	156 (8.8%)	14 (8.4%)	16 (6.1%)	0.353

ESSW, emergency short-stay ward; EM, emergency medicine; GW, general ward

In the multivariable linear regression analysis, the ESSW-EM group was independently associated with shorter ED length of stay compared with both the ESSW-Other group (adjusted P < 0.001, Table 4) and the GW group (adjusted P < 0.001, Table 4). In the multivariable logistic regression analyses, the ESSW-EM group was independently associated with lower hospital mortality when compared with both the ESSW-Other group (adjusted P = 0.030, Table 5) and the GW group (adjusted P < 0.001; Table 5).

**Table 4:** Multivariable linear regression analysis for emergency department stay length

	Adjusted coefficient	95% confidence interval		*P* value
KTAS level				
KTAS 1	Reference			
KTAS 2	1.79	0.97	2.61	< 0.001
KTAS 3	1.88	1.03	2.73	< 0.001
KTAS 4	1.47	0.55	2.38	0.002
KTAS 5	-0.49	-1.87	0.89	0.488
Visit route				
Direct visit	Reference			
Via outpatient clinic	-0.27	-0.57	0.03	0.075
Via outside hospital	0.17	-0.07	0.41	0.169
Others	-2.30	-8.91	4.30	0.494
Type of visit (medical)	2.19	1.72	2.66	< 0.001
Systolic blood pressure				
90–120 mmHg	Reference			
< 90 mmHg	1.47	0.66	2.28	< 0.001
> 120 mmHg	-0.48	-0.72	-0.24	< 0.001
Heart rate				
50–100 beats per min	Reference			
< 50 beats per min	-0.25	-0.77	0.28	0.356
> 100 beats per min	0.77	0.54	1.01	< 0.001
Respiratory rate, by 1 breath per min increment	0.06	0.03	0.09	< 0.001
Body temperature				
36–38 °C	Reference			
< 36 °C	-0.73	-1.34	-0.12	0.019
> 38 °C	0.79	0.44	1.15	< 0.001
SpO2, by 1% increment	-0.02	-0.05	0.02	0.319
Patient response				
Alert	Reference			
Response to verbal stimulus	1.37	0.92	1.83	< 0.001
Response to pain	1.57	0.61	2.53	0.001
Unresponsive	-0.60	-2.55	1.35	0.548
Year				
2017	Reference			
2018	-0.88	-1.13	-0.64	< 0.001
2019	-1.90	-2.15	-1.66	< 0.001
Group				
ESSW-EM	Reference			
ESSW-Other	1.08	0.70	1.46	< 0.001
GW	3.35	3.12	3.57	< 0.001

KTAS, Korean Triage and Acuity Scale; SpO2, peripheral capillary oxygen saturation; ESSW, emergency short-stay ward; EM, emergency medicine; GW, general ward

**Table 5:** Multivariable logistic regression analysis for 28-day hospital mortality

	Adjusted odds ratio	95% confidence interval		*P* value
Age	1.02	1.01	1.02	< 0.001
Male sex	1.32	1.14	1.53	< 0.001
KTAS level				
KTAS 1	Reference			
KTAS 2	0.74	0.53	1.03	0.078
KTAS 3	0.43	0.30	0.62	< 0.001
KTAS 4	0.17	0.09	0.32	< 0.001
KTAS 5	0.32	0.10	1.07	0.065
Visit route				
Direct visit	Reference			
Via outpatient clinic	1.04	0.84	1.30	0.714
Via outside hospital	0.08	1.68		1.202
Others	10.62	1.07	105.28	0.044
Type of visit (medical)	4.56	2.02	10.33	< 0.001
Systolic blood pressure				
90–120 mmHg	Reference			
< 90 mmHg	1.31	0.94	1.84	0.115
> 120 mmHg	0.41	0.35	0.48	< 0.001
Heart rate				
50–100 beats per min	Reference			
< 50 beats per min	0.42	0.22	0.80	0.009
> 100 beats per min	2.73	2.33	3.20	< 0.001
Respiratory rate, by 1 breath per min increment	1.05	1.03	1.06	< 0.001
Body temperature				
36–38 °C	Reference			
< 36 °C	0.82	0.54	1.25	0.355
> 38 °C	0.50	0.38	0.64	< 0.001
SpO2, by 1% increment	0.98	0.97	0.99	0.003
Missing SpO2 value	0.61	0.49	0.76	< 0.001
Patient response				
Alert	Reference			
Response to verbal stimulus	1.82	1.44	2.29	< 0.001
Response to pain	1.46	0.93	2.28	0.100
Unresponsive	2.06	0.97	4.37	0.060
Group				
ESSW-EM	Reference			
ESSW-Other	1.33	0.96	1.85	0.089
GW	2.95	2.45	3.55	< 0.001

KTAS, Korean Triage and Acuity Scale; SpO2, peripheral capillary oxygen saturation; ESSW, emergency short-stay ward; EM, emergency medicine; GW, general ward

**Fig. 3:**
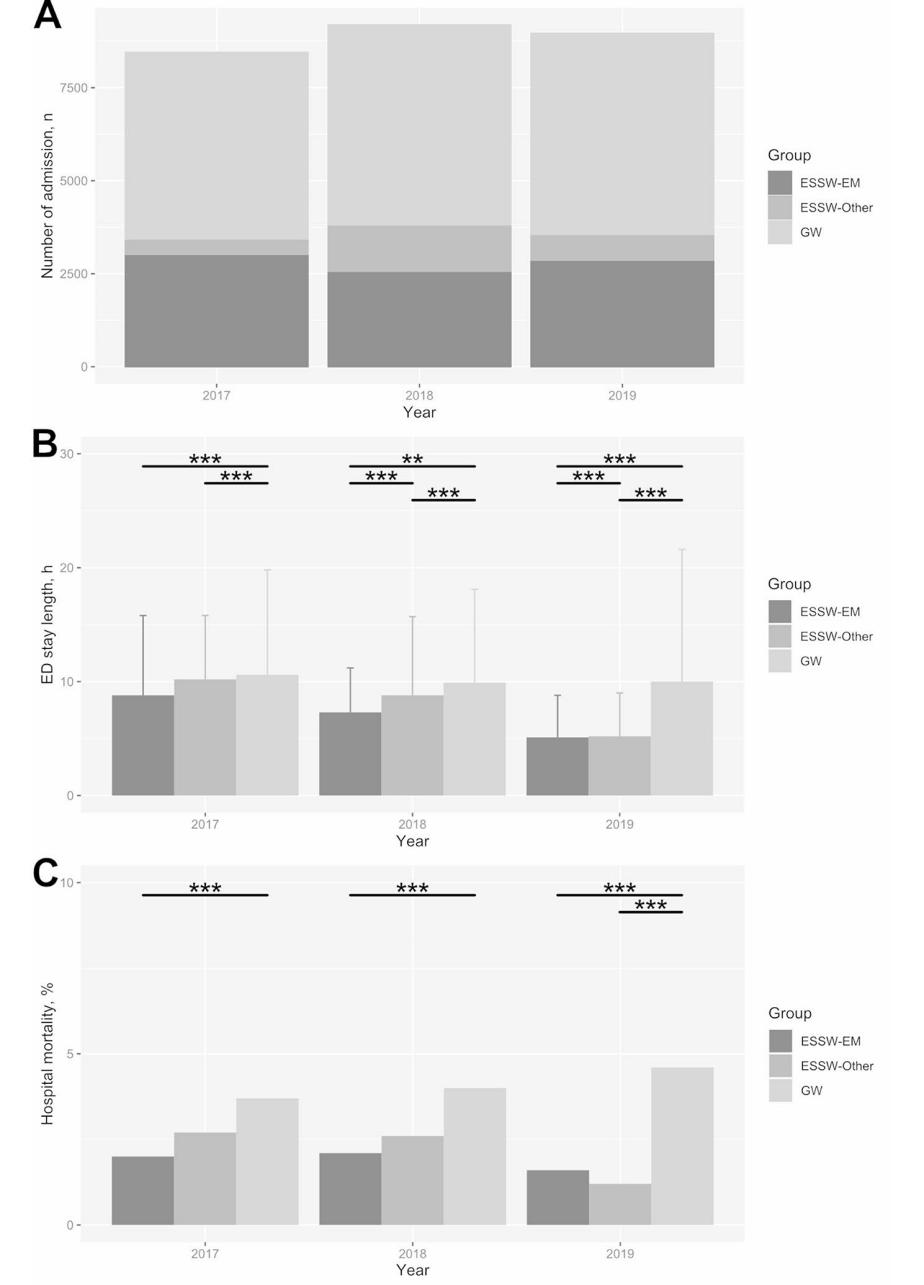
Distribution, ED stay length and hospital mortality according to the study group and visit year **3A**: distribution of the participants according to the study group and visit year; **3B**: ED stay length according to the study group and visit year; **3C**: hospital mortality according to the study group and visit year ^**^P < 0.01; ^***^P < 0.001 ED, emergency department; EM, emergency medicine; ESSW, emergency short-stay ward; GW, general ward

In the subgroup analysis with subgroups divided according to ED visit year (Fig. 3A), both ED length of stay (Fig. 3B) and hospital mortality (Fig. 3C) were significantly different among patient groups in each subgroup (P < 0.001 for both ED length of stay and hospital mortality in all year subgroups). In the post hoc analysis, ED length of stay of the ESSW-EM group was significantly shorter than that of the ESSW-Other group in subgroups 2018 (adjusted P = 0.008) and 2019 (adjusted P < 0.001). ED length of stay of the ESSW-EM group was shorter than that of the GW group in subgroups 2017, 2018 and 2019 (adjusted P < 0.001 in all subgroups). Hospital mortality did not show significant difference between the ESSW-EM group and the ESSW-Other group in subgroups 2017, 2018 and 2019 (P = 1.000, P = 0.918 and P = 1.000, respectively), while it was lower in the ESSW-EM group compared with that in the GW group in subgroups 2017, 2018 and 2019 (P < 0.001 in all subgroups).

**Fig. 4:**
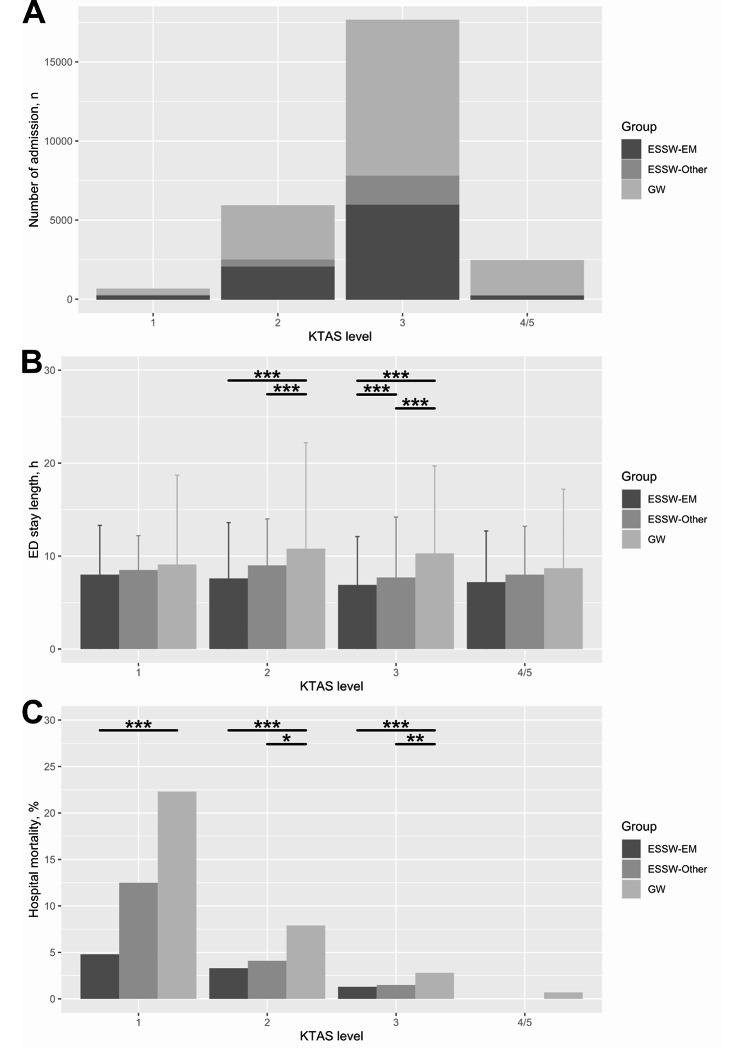
Distribution, ED stay length and hospital mortality according to the study group and KTAS level **4A**: distribution of the participants according to the study group and KTAS level; **4B**: ED stay length according to the study group and KTAS level; **4C**: hospital mortality according to the study group and KTAS level ^*^P < 0.05; ^**^P < 0.01; ^***^P < 0.001 ED, emergency department; EM, emergency medicine; ESSW, emergency short-stay ward; GW, general ward; KTAS, Korean Triage and Acuity Scale

In the subgroup analysis stratified with KTAS level (Fig. 4A), significant differences were found in ED length of stay among study groups in KTAS 2 and KTAS 3 subgroups (P < 0.001 for each; Fig. 4B). Hospital mortality showed significant difference among study groups in all KTAS subgroups, except the KTAS 4 and 5 subgroup (P < 0.001 for each; Fig. 4C). In the post hoc analysis, ED length of stay of the ESSW-EM group was significantly shorter than that of the ESSW-Other group in the KTAS 3 subgroup (adjusted P < 0.001) and was shorter than that of GW group in the KTAS 2 and the KTAS 3 subgroups (adjusted P < 0.001 for each). Hospital mortality was also significantly lower in ESSW-EM group than that of GW group in the KTAS 1, the KTAS 2 and the KTAS 3 subgroups (adjusted P < 0.001 for each).

## Discussion

In this study, we found that admission to the ESSW operated by EM physician significantly reduced ED length of stay compared with admission to both the ESSW operated by other physicians and the GW. Moreover, hospital mortality of patients admitted to the ESSW on day 28 was lower than that of who were admitted to the GW. The finding was similar even when the patients were stratified by year: ED length of stay of ESSW-EM group was consistently shorter than that of the ESSW-Other and the GW groups, except that the ED length of stay in 2017 was not different between the ESSW-EM and the ESSW-Other groups. Hospital mortality on day 28 in the ESSW-EM group was lower than that in the GW group in all the year subgroups.

ED crowding has known to be associated with worse clinical outcomes. ED length of stay is often used as a surrogate for the degree of ED crowding [12]. Previous studies have reported that the longer time spent in ED, the worse clinical outcomes such as inpatient length of stay, time-to-treatment, and mortality [13, 14]. Based on these results, one can presume that admission to the ESSW under the care of EM may be effective to alleviate ED burden and improve subsequent patient outcomes.

As the study hospital has been a tertiary academic hospital operating a regional emergency medical center since 2004, the hospital policy allows patients to stay up to three days at maximum in the ESSW. As consequence, a large proportion of the patients admitted to the ESSW had to be transferred out after a short period of acute care, and the ESSW-EM group showed higher transfer rate than that of other groups. Several studies reported the association interhospital transfer with adverse clinical outcome including higher ICU and hospital utilization, lower frequency of discharges home and increased mortality [15, 16]. However, Hill et al. reported that there was no difference in mortality between transfer and direct admission in major trauma patients [17]. Therefore, the impact on clinical outcome of interhospital transfer remains controversial yet. Although the clinical outcomes, especially 28-day mortality, of the patients transferred out was not traced in this study, 28-day hospital mortality was lower in the ESSW-EM group. The rate of ED revisit within 7 days from transfer out was similar among the three groups and it was as low as approximately 2%, which indicates that probability of clinical deterioration are low in this patient group. In this context, the results of the present study suggest that the ESSW operated by EM physicians reduced ED burden without cost of clinical outcomes.

As far as we know, this study was the first study which investigated effectiveness of the ESSW operated by EM physicians. This study suggested that the EM physicians are capable of caring for hospitalized patients, which contributes the basis for the expansion of ESSW operation by EM physicians.

The present study has several limitations. First, owing to the retrospective nature of the study, we could not include confounding factors such as main diagnosis, disease severity scores and comorbidity status which might have been associated with clinical outcomes and might have affected the decision for admission to the ESSW. Moreover, the study result was derived from a single hospital data, which limited the generalizability of the results. Additionally, a before-and-after comparison of the study outcomes might enhance the clinical utility of the study. However, for the ESSW has been operated since 2004 in the study hospital and clinical environment has been changed since then, it is not feasible to implement the before-and-after design in 2022. Another limitation of this study is that the proportion of the transferred-out patients were substantially higher in the ESSW-EM group than in the other groups. The difference in the transferring rate might have led to a biased result, and it was also difficult to compare precise short-term and long-term outcomes. However, the similarly low ED revisit rate indirectly implies that the fatal short-term clinical outcomes of the patients transferred out might be infrequent. Finally, although we did not include detailed clinical information such as diagnosis, laboratory results or treatment, KTAS and vital signs might reflect the clinical severity of the patients.

## Conclusions

Admission to the ESSW and treatment by EM physicians was independently associated with both shorter emergency department length of stay and lower hospital mortality compared with both admission to the ESSW by physicians of other specialties and admission to the general wards in the adult patients visiting an emergency department.

## Data Availability

The datasets used and/or analysed during the current study are available from the corresponding author on reasonable request.

## List of Abbreviations

EM: Emergency medicine
ED: Emergency department
ESSW: Emergency short-stay ward
GW: General wards
KTAS: Korean Triage and Acuity Scale
ICU: Intensive care unit

## References

[1] Singer AJ, Thode HC, Viccellio P, Pines JM (2011). The Association between length of Emergency Department Boarding and Mortality. Acad Emerg Med.

[2] Geelhoed GC, de Klerk NH (2012). Emergency department overcrowding, mortality and the 4-hour rule in western Australia. Med J Aust.

[3] Jones P, Schimanski K (2010). The four hour target to reduce emergency department ‘waiting time’: a systematic review of clinical outcomes. Emerg Med Australasia.

[4] Jones PG, Olsen S (2011). Point prevalence of access block and overcrowding in New Zealand emergency departments in 2010 and their relationship to the ‘Shorter stays in ED’ target. Emerg Med Australasia.

[5] Yoon BS, Choa MH, Kong TY, Joo YS, Ko DR, Hwang YJ (2018). The effect of time target on overcrowding and clinical quality in the ED: a systematic review and meta-analysis. J Korean Soc Emerg Med.

[6] Bartiaux M, Mols P (2017). [Evaluations by hospital-ward physicians of patient care management quality for patients hospitalized after an emergency department admission]. Rev Med Brux.

[7] Cirillo W, Freitas LRC, Kitaka EL, Matos-Souza JR, Silva MR, Coelho OR (2021). Impact of emergency short-stay unit opening on in-hospital global and cardiology indicators. J Eval Clin Pract.

[8] Galipeau J, Pussegoda K, Stevens A, Brehaut JC, Curran J, Forster AJ (2015). Effectiveness and safety of short-stay units in the emergency department: a systematic review. Acad Emerg Med.

[9] Ok M, Choi A, Kim MJ, Roh YH, Park I, Chung SP (2020). Emergency short-stay wards and boarding time in emergency departments: a propensity-score matching study. Am J Emerg Med.

[10] Shetty AL, Teh C, Vukasovic M, Joyce S, Vaghasiya MR, Forero R (2017). Impact of emergency department discharge stream short stay unit performance and hospital bed occupancy rates on access and patient flowmeasures: a single site study. Emerg Med Australas.

[11] Strøm C, Mollerup TK, Kromberg LS, Rasmussen LS, Schmidt TA (2017). Hospitalisation in an emergency department short-stay unit compared to an internal medicine department is associated with fewer complications in older patients - an observational study. Scand J Trauma Resusc Emerg Med.

[12] Badr S, Nyce A, Awan T, Cortes D, Mowdawalla C, Rachoin JS (2022). Measures of Emergency Department Crowding, a systematic review. How to make sense of a long list. Open access emergency medicine: OAEM.

[13] Burgess L, Ray-Barruel G, Kynoch K (2022). Association between emergency department length of stay and patient outcomes: a systematic review. Res Nurs Health.

[14] Salehi L, Phalpher P, Valani R, Meaney C, Amin Q, Ferrari K (2018). Emergency department boarding: a descriptive analysis and measurement of impact on outcomes. Cjem.

[15] Baig SH, Gorth DJ, Yoo EJ. Critical Care Utilization and Outcomes of Interhospital Medical Transfers at Lower Risk of Death. 0(0):08850666211022613. 10.1177/08850666211022613 34080443

[16] Sokol-Hessner L, White AA, Davis KF, Herzig SJ, Hohmann SF (2016). Interhospital transfer patients discharged by academic hospitalists and general internists: characteristics and outcomes. J Hosp Med.

[17] Hill AD, Fowler RA, Nathens AB (2011). Impact of interhospital transfer on outcomes for trauma patients: a systematic review. J trauma.

